# Comparative Effectiveness of Amisulpride and Clozapine in the Treatment of Schizophrenia: A Systematic Review and Meta-Analysis

**DOI:** 10.7759/cureus.62625

**Published:** 2024-06-18

**Authors:** Rommy Cedeno, Arturo P Jaramillo, Ahmad R Khan

**Affiliations:** 1 Molecular Pharmacology and Therapeutics in Psychiatry, Columbia University, New York, USA; 2 General Practice, Universidad Estatal de Guayaquil, Machala, ECU; 3 Psychiatry, Carilion Clinic, Roanoke, USA

**Keywords:** antipsychotic medication, augmentation therapy, schizophrenia, amisulpride, clozapine

## Abstract

In approximately one-third of individuals with schizophrenia, the illness demonstrates a poor response to standard antipsychotic treatments. Although a relatively small proportion fails to achieve remission after the initial exposure to either first- or second-generation antipsychotic drugs, the condition often becomes progressively more resistant to medication following subsequent relapses. We conducted comprehensive searches in databases such as PubMed and PubMed Central, extracting and assessing data quality using the Cochrane risk-of-bias tool for randomized clinical trials (RCTs). A random effects model was employed to calculate the pooled prevalence and explore heterogeneity, utilizing the I^2^ statistic. Subgroup analyses differentiated between experimental and placebo groups, while sensitivity analyses assessed the robustness of our findings, and publication bias was examined. Our meta-analysis included a sample size of 323 patients from seven studies out of the 10 selected articles. The pooled sample evaluated the effectiveness of amisulpride and clozapine in treating schizophrenia, with Positive and Negative Syndrome Scale (PANSS)-positive and PANSS-negative scores used in the subgroup analysis. The analysis revealed a heterogeneity of 78% and a statistically significant p-value of <0.05, favoring amisulpride and clozapine for treating schizophrenia either as monotherapy or in combination. These findings indicate that the effectiveness of these drugs is statistically significant. Our study underscores the necessity of conducting larger RCTs to further elucidate the optimal dosage and guideline criteria for prescribing amisulpride, clozapine, or their combination for patients resistant to first- and second-generation antipsychotics.

## Introduction and background

For approximately 20%-30% of individuals with schizophrenia, the illness does not respond to two or more adequate trials, in terms of dose and duration, of first-line antipsychotic medications. This condition is clinically defined as treatment-resistant schizophrenia (TRS), which is linked to significantly diminished quality of life for patients and substantially higher socioeconomic costs compared to non-TRS, imposing a considerable burden on both individuals and society [1-3]. Since 1988, clozapine has been endorsed as the gold standard treatment for TRS in all clinical guidelines. Meta-analyses have consistently shown that clozapine outperforms first-generation antipsychotics in managing overall symptoms, and it is superior to both first- and second-generation antipsychotics in addressing total, positive, and negative symptoms [4,5]. Nevertheless, up to 60% of patients treated with clozapine do not experience adequate symptom relief, and the optimal clinical management for these patients remains unclear.

Clozapine-resistant schizophrenia (CRS) is defined by the Treatment Response and Resistance in Psychosis (TRRIP) Working Group as the persistence of positive, negative, or cognitive symptoms of at least moderate severity after an adequate trial of clozapine [6]. Specifically, persistent positive or negative symptoms are defined as having two or more symptoms in the respective domain of at least moderate severity, or one symptom rated as severe. While cognitive impairment is a prominent feature of schizophrenia, the TRRIP guidelines do not specifically define cognitive symptoms [7]. One of the most critical questions in managing schizophrenia is how to treat CRS effectively. Typically, this condition shows minimal symptomatic improvement from the baseline observed before clozapine treatment, with ongoing functional deficits and disabling symptoms [7].

The TRRIP Working Group recommends offering clozapine treatment for at least three months after achieving therapeutic plasma levels for TRS, but it does not provide strategies for managing persistent symptoms despite adequate clozapine monotherapy [7]. Evidence from meta-analyses suggests only marginal or low-quality benefits for pharmacological strategies that combine clozapine with other treatments after an insufficient response to clozapine monotherapy [8,9]. Randomized controlled trials (RCTs) investigating pharmacological options for CRS have been included in meta-analyses, but these RCTs exhibit significant methodological heterogeneity [8,10]. High-level evidence is further complicated by varying or absent definitions of CRS.

Amisulpride is often regarded as a favorable option for clozapine augmentation therapy due to its perceived advantages in tolerability and safety, particularly concerning extrapyramidal side effects, weight gain, and metabolic side effects [10]. This perception may contribute to its relatively frequent use in clinical practice in the UK for augmenting clozapine, despite the current lack of robust clinical evidence regarding the potential risks and benefits of this drug combination [11]. Additionally, the selective dopamine D2/D3 blocking properties of amisulpride are thought to provide a complementary receptor profile to clozapine, which may further explain its popularity as an augmentation strategy [10-12].

Electroconvulsive therapy (ECT) has shown efficacy in treating clozapine-refractory positive symptoms in open-label studies, but more high-quality trials are necessary before ECT can be routinely included in treatment algorithms [11,12]. A recent RCT involving 487 participants with CRS did not find a benefit for clozapine augmentation with cognitive-behavioral therapy [13]. Despite limited evidence of effectiveness, antipsychotic polypharmacy is prevalent among patients with schizophrenia, with clozapine often combined with another antipsychotic in up to half of clozapine prescriptions [13]. This frequent co-treatment reflects the need for comprehensive guidelines that outline a hierarchy of pharmacological and nonpharmacological treatment recommendations for CRS patients [13].

Additionally, treatment with clozapine is often delayed due to barriers related to prescribers and institutions, reducing the likelihood of a potential treatment response [13]. Given the limited evidence available for managing CRS and the significant challenges it poses for clinicians, a two-step survey and consensus process among international experts was initiated to develop meaningful treatment options for CRS patients with persistent symptoms despite adequate clozapine monotherapy [14]. Such consensus approaches have been previously employed to establish antipsychotic dosing and recovery definitions in psychosis. This systematic review and meta-analysis aim to thoroughly assess the most recent studies from the past decade regarding the effectiveness of amisulpride and clozapine in treating schizophrenia. One of the primary objectives is to provide a comprehensive evaluation of these treatments to enhance clinical understanding and improve patient outcomes.

## Review

Methods

Review Records and Search for Studies

This systematic review adhered to the guidelines of the Preferred Reporting Items for Systematic Reviews and Meta-Analyses (PRISMA) [15]. The article selection process involved independent researchers conducting comprehensive searches in PubMed, PubMed Central, and other databases. Details of the search methodology employed can be found in Table 1.

**Table 1:** Search strategy for databases

Search Strategy	Databases Used	Number of Papers Identified
Schizophrenia agents AND Antipsychotics AND Amisulpride AND Clozapine	PubMed	447
( "Amisulpride/administration and dosage"[Majr] OR "Amisulpride/therapeutic use"[Majr] )) AND ( "Clozapine/administration and dosage"[Majr] OR "Clozapine/therapeutic use"[Majr] )	PubMed Central	2,314
"Schizophrenia agents [tw]" AND "Antipsychotics [tiab]" AND Amisulpride [all]"	Others	83

Inclusion and Exclusion Criteria

Two independent authors utilized the Covidence software to screen the search results obtained from two databases following pre-established inclusion and exclusion criteria, as shown in Table 2.

**Table 2:** Inclusion and exclusion criteria

Inclusion	Exclusion
Free, full text on antipsychotics focused more on clozapine and amisulpride	Articles that include pregnant woman
Articles from the past 10 years	Articles from 2013 and below
English-language articles	Non-English studies
Prospective or retrospective studies	Case reports
Human trials	Animal trials

Data Extraction

During a thorough analysis of the relevant research, several significant findings emerged. The design of each trial, the number of individuals given clozapine, amisulpride, or the augmentation of amisulpride in clozapine patients, and the findings observed in both the experimental and placebo cohorts are among these significant findings.

Risk-of-Bias Assessment

For the purpose of determining whether or not the studies that were chosen for our investigation had any possible biases, we used the Cochrane risk-of-bias tool, which was developed especially for RCTs. In the process of assessing the quality of case-series research, this instrument has garnered widespread recognition for its efficiency [16]. Reviewers impartially evaluated the potential for bias in each research and resolved any discrepancies in their assessments through in-depth conversations.

Statistical analysis

We used RevMan version 5.4 (2020, The Cochrane Collaboration, The Nordic Cochrane Centre, Copenhagen, Denmark) for all statistical analyses. We expressed the trial results using the mean difference and 95% confidence intervals and pooled the data using an odds ratio effects model. We followed Mantel-Haenszel et al.'s methodology to calculate the standard deviations or standard errors not reported in the trials. We chose a fixed-effect model over a random-effect model due to the potential high variance arising from diverse study designs and populations.

We used forest plots to visually assess the pooled results. We used the chi-square test to identify any discrepancies between the subgroups. We quantified study heterogeneity using Higgins I^2^. A visual examination of the funnel plot was used to evaluate publication bias, with a significance threshold set at p<0.05.

Results

**Figure 1:**
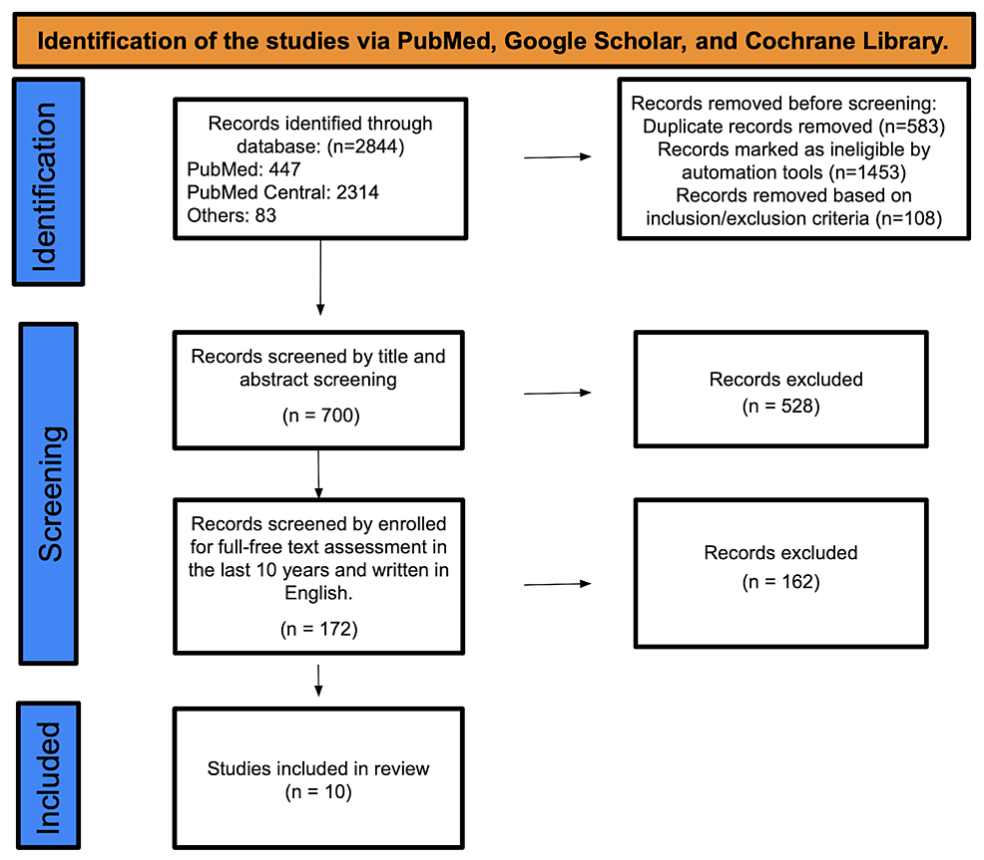
PRISMA diagram PRISMA, Preferred Reporting Items for Systematic Reviews and Meta-Analyses

We searched PubMed, PubMed Central, and other databases and found a total of 2,844 studies. Based on inclusion and exclusion criteria, we marked 108 studies as ineligible, removed 583 duplicate studies, and selected 1453 studies through the automation tool. We screened 700 studies for title and abstract, discarding 528 as they did not align with our study's purpose. We selected the remaining 172 papers based on their English content and full-free text evaluation from the previous ten years, eliminating 162 studies and enlisting only 10 for the final data collection (Figure 1).

Table 3 provides an in-depth description of the articles we decided to use.

**Table 3:** Data extraction CGI, Clinical Global Impression; CGI-CB, Clinical Global Impressions-Clinical Benefit; CGI-S, CGI-Severity; CRS, clozapine-resistant schizophrenia; FES, first-episode schizophrenia; PANSS, Positive and Negative Syndrome Scale; RCT, randomized clinical trial; TRRIP, Treatment Response and Resistance in Psychosis; SANS, Scale for the Assessment of Negative Symptoms; SAPS, Scale for the Assessment of Positive Symptoms; SCoRS, Schizophrenia Cognition Rating Scale; SMART-CAT, Sequential Multiple-Assignment Randomized Trials comparing Antipsychotic Treatments; TRS, treatment-resistant schizophrenia

Author	Year of Publication	Study Design	Research Methodology	Overcome Evaluation
Wagner et al. [13]	2020	RCT	They polled members of TRRIP Working Group online.	This consensus-based set of suggestions helps manage this problematic clinical scenario, given the inadequate data from RCT of CRS treatment methods.
Stabell et al. [17]	2023	RCT	Throughout the year, 144 patients with FES or multi-episode schizophrenia spectrum disorder underwent eight evaluations.	In the long-term phase (weeks 6–52), amisulpride and olanzapine increased insight beyond psychotic symptom reduction.
Kjelby et al. [18]	2023	RCT	We used latent growth curve modeling for schizophrenia-psychotic individuals to assess their success.	At the 12-month follow-up, all treatment groups reduced depressive symptoms, although there were no statistically significant differences.
Alisauskiene et al. [19]	2023	RCT	The PANSS was used to measure clinical symptoms.	The three examined antipsychotics did not significantly reduce PANSS-positive subscale scores in individuals with or without drug use. Older drug users treated with amisulpride had a more significant PANSS-positive subscale score drop than younger patients.
Zhu et al. [20]	2022	RCT	We randomly assigned 80 individuals to receive either clozapine, amisulpride, or a placebo.	The amisulpride group showed a greater treatment response rate (P = 0.04) and lower CGI severity and efficacy ratings at weeks 6 and 12 compared to the placebo group (P< 0.05).
Li et al. [21]	2021	RCT	Nine Chinese universities participated in SMART-CAT, an RCT multicenter experiment. This research tracked 720 FES participants for 12 months.	The SMART-CAT study supported antipsychotic selection for FES patients who failed the first trial.
Barnes et al. [22]	2018	RCT	68 individuals with TRS and persistent symptoms after a specific trial participated in a 12-week RCT of clozapine augmentation with amisulpride.	Amisulpride-treated individuals were more likely to meet the clinical response threshold and reduce negative symptoms, although these improvements were not statistically significant and only appeared at 12 weeks.
Barnes et al. [23]	2017	RCT	Participants were 18–65-year-olds with TRS who were unresponsive. Participants received 400 mg of amisulpride or two placebo capsules for the first 4 weeks and then may titrate to 800 mg or four placebo capsules for the final 8 weeks.	There were 68 randomly selected participants. By 12-week follow-up, amisulpride patients had a higher likelihood of responding and a bigger reduction in negative symptoms than placebo patients, although neither outcome was statistically significant.
Kim et al. [24]	2016	RCT	Six weeks with schizophrenic patients. At week 6, the main endpoint was CGI-CB-based clinical benefit improvement.	Out of 37 patients converted to amisulpride, 76% completed the trial and 56.8% achieved CGI-CB clinical benefit. Week 6 CGI-S values improved significantly from baseline.
Kumar et al. [25]	2014	RCT	For the research, 40 adult schizophrenia inpatients with informed consent met inclusion/exclusion criteria.	The SANS, SAPS, SCoRS interviewer, and SCoRS global scores improved by 74.96%, 13.36%, 54.14%, and 42.00%, respectively, in the amisulpride group.

Meta-Analysis of Outcomes

**Figure 2:**
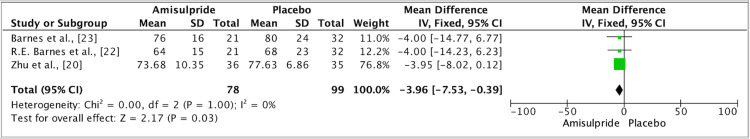
Forest plot for studies on the efficacy of amisulpride versus placebo. [20,22,23]

The results of three studies indicated that amisulpride was more effective than placebo, with a mean difference of -3.96. This mean difference was calculated using a fixed-effect model and had a 95% confidence interval ranging from -7.53 to -0.39. The p-value was 1.00, and there was no heterogeneity (I² = 0%) (Figure 2).

**Figure 3:**
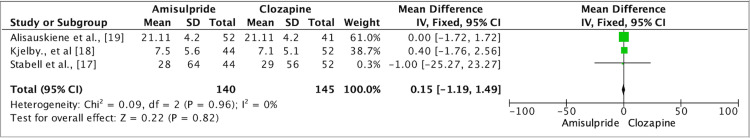
A forest plot for studies on amisulpride augmentation in the clozapine-unresponsive schizophrenia group. [17-19]

The results of three studies showed a mean difference of 0.15 in amisulpride augmentation for patients with schizophrenia who did not respond to clozapine. The mean difference was 0.15 with a fixed effect, a 95% confidence interval ranging from -1.19 to 1.49, a p-value of 0.96, and no heterogeneity (I² = 0%) (Figure 3).

**Figure 4:**
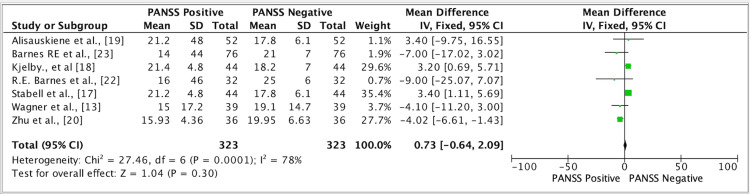
Forest plot for studies on the overall efficacy of amisulpride and clozapine medication through PANSS-positive and PANSS-negative groups. PANSS, Positive and Negative Syndrome Scale [13,17-20,22,23]

The results of seven studies showed a mean difference of 0.73 in the efficacy of amisulpride and clozapine medication through PANSS-positive and PANSS-negative scores. The mean difference was 0.73 (fixed effect), 95% CI was -0.64, 2.09, the p-value was <0.0001, and the heterogeneity (I^2^) was 78% (Figure 4).

**Figure 5:**
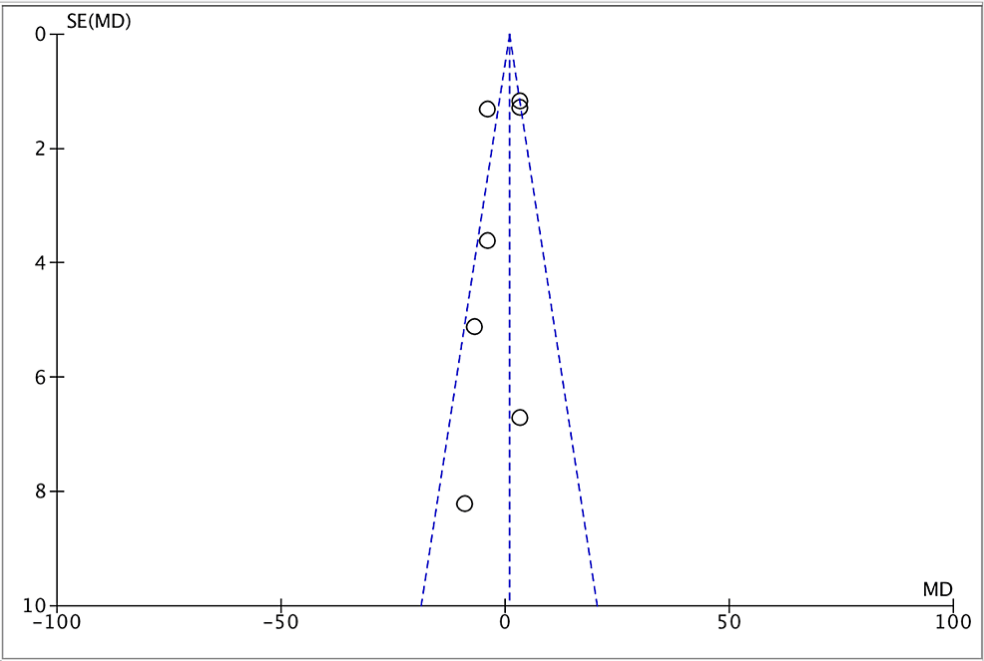
Funnel plot for all included studies about the overall efficacy of amisulpride and clozapine medication through PANSS=positive and PANSS-negative groups. PANSS, Positive and Negative Syndrome Scale [13,17-20,22,23]

Publication bias was seen in four studies (Figure 5).

Discussion

In our meta-analysis, clozapine stands out as the only antipsychotic medication with strong evidence supporting its efficacy in strictly defined TRS. However, even with clozapine, only 30%-60% of patients experience an adequate response. To enhance its efficacy, clinicians often add another antipsychotic to the regimen, though this strategy typically yields only modest benefits.

For instance, a multi-center, double-blind comparison evaluated two dosage levels of amisulpride (100 mg/day and 300 mg/day) against a placebo over six weeks. The study revealed significant differences between the placebo and amisulpride groups, with the mean total Scale for the Assessment of Negative Symptoms (SANS) reducing by 22.8% in the placebo group, 40.6% in the 100 mg/day group, and 45.9% in the 300 mg/day group.

Our comprehensive review synthesizes data from various studies, presenting a detailed analysis of clozapine and amisulpride as monotherapies, as well as the augmentation of amisulpride in patients already taking clozapine. The study by Li et al. also explained the Sequential Multiple-Assignment Randomized Trials comparing Antipsychotic Treatments (SMART-CAT) for schizophrenia [21]. It included the study's background, goals, and design. The goal of this trial is to find the best way to treat schizophrenia in its early stages. It will look at important issues like when to start taking clozapine and how well it works compared to other second-generation antipsychotics (SGAs) for people who do not respond to their first antipsychotic [21]. The SMART design, which combines sequential and dynamic therapy, is perfect for testing treatment options in real-life clinical settings. It helps doctors figure out the best ways to treat people with their first episode of schizophrenia [21].

Stabell et al.'s RCT was the first to investigate the differential effects of antipsychotics on insight in a mixed sample of antipsychotic-naïve and previously medicated patients [17]. They found that all study drugs improved insight by reducing overall psychotic symptoms. In particular, amisulpride and olanzapine improved insight on top of reducing general symptoms. There were no significant differences between patients who had never taken an antipsychotic drug before and those who had [17].

Kjelby et al. reported a significant reduction in depressive symptoms in patients with current psychosis within the schizophrenia spectrum treated with amisulpride, with the steepest improvement occurring in the first six weeks [18]. Another RCT by Kim et al. showed that patients who switched from atypical antipsychotics such as clozapine and olanzapine to amisulpride had better clinical benefits in terms of effectiveness and tolerability. This suggests that amisulpride could be a good alternative for patients who are not getting the best results from their current antipsychotics [24].

A study by Zhu et al. showed that amisulpride augmentation could safely improve clinical symptoms and cognitive function in schizophrenia patients who were not responding to clozapine treatment. At weeks 6 and 12, the amisulpride group had significantly better positive and general psychopathological symptoms compared to the placebo group [20]. Furthermore, the clinical global impression severity and clinical global efficacy scores were significantly better in the amisulpride augmentation group [20].

Kumar et al.'s RCT showed that amisulpride treatment significantly improved negative symptoms and cognitive impairments in schizophrenia patients [25]. Alisauskiene et al. found that drug use does not significantly affect the overall effectiveness of amisulpride, aripiprazole, and olanzapine in patients with schizophrenia spectrum disorders, suggesting that all patients, regardless of drug use, should receive these pharmacological treatments [19]. However, amisulpride may be particularly suitable for older patients with drug use [19].

According to Luykx et al., a large nationwide cohort study showed that clozapine and oral olanzapine are consistently linked with better outcomes, including lower risks of psychiatric ward readmission, treatment failure, and death, compared to people who did not use antipsychotics or other antipsychotics [14]. Their findings recommend reconsidering clozapine for patients who have previously received it [14].

Some studies revealed no statistical significance or a poor response to monotherapy or a combination of therapies. We have Wagner et al.'s RCT, which highlighted the challenge of managing patients who do not respond to clozapine, underscoring the need for further research [13]. The RCT by Barnes et al. found that amisulpride augmentation was linked to small improvements in negative symptoms and a slightly higher chance of overall symptom reduction within 12 weeks, but these differences were not significant at the statistical level [23]. Despite amisulpride's favorable tolerability profile, combining it with clozapine was associated with a greater side effect burden, including cardiac symptoms. The risk-benefit profile of amisulpride augmentation for CRS warrants further investigation in larger studies [22].

## Conclusions

The augmentation of clozapine with another antipsychotic, such as amisulpride, shows modest benefits but is commonly practiced. Our study comparing different dosages of amisulpride revealed significant improvements in negative symptoms, suggesting its potential as a beneficial adjunct therapy. The findings also underline the complexities and unanswered questions in optimizing sequential antipsychotic treatments, particularly in first-episode schizophrenia. Amisulpride, with its favorable efficacy and tolerability, may offer a valuable alternative for patients not responding adequately to other antipsychotics. However, the risk-benefit profile, especially concerning side effects, warrants further investigation. Overall, this comprehensive review and analysis underscore the importance of personalized treatment strategies and continuous research to enhance therapeutic outcomes for schizophrenia patients.
